# Quantitative pupillometry and radiographic markers of intracranial midline shift: A pilot study

**DOI:** 10.3389/fneur.2022.1046548

**Published:** 2022-12-06

**Authors:** Ivy So Yeon Kim, Oluwafemi O. Balogun, Brenton R. Prescott, Hanife Saglam, DaiWai M. Olson, Kinley Speir, Sonja E. Stutzman, Nathan Schneider, Veronica Aguilera, Bethany L. Lussier, Stelios M. Smirnakis, Josée Dupuis, Asim Mian, David M. Greer, Charlene J. Ong

**Affiliations:** ^1^Boston University School of Medicine, Boston, MA, United States; ^2^Boston Medical Center, Boston, MA, United States; ^3^Mass General Brigham, Boston, MA, United States; ^4^Harvard Medical School, Boston, MA, United States; ^5^University of Texas Southwestern Medical Center, Dallas, TX, United States; ^6^Jamaica Plain Veterans Administration Medical Center, Boston, MA, United States; ^7^Boston University School of Public Health, Boston, MA, United States; ^8^Department of Epidemiology, Biostatistics and Occupational Health, McGill University, Montreal, QC, Canada

**Keywords:** pupillometry, neurocritical care, herniation, radiology, midline shift

## Abstract

**Background:**

Asymmetric pupil reactivity or size can be early clinical indicators of midbrain compression due to supratentorial ischemic stroke or primary intraparenchymal hemorrhage (IPH). Radiographic midline shift is associated with worse functional outcomes and life-saving interventions. Better understanding of quantitative pupil characteristics would be a non–invasive, safe, and cost-effective way to improve identification of life-threatening mass effect and resource utilization of emergent radiographic imaging. We aimed to better characterize the association between midline shift at various anatomic levels and quantitative pupil characteristics.

**Methods:**

We conducted a multicenter retrospective study of brain CT images within 75 min of a quantitative pupil observation from patients admitted to Neuro-ICUs between 2016 and 2020 with large (>1/3 of the middle cerebral artery territory) acute supratentorial ischemic stroke or primary IPH > 30 mm^3^. For each image, we measured midline shift at the septum pellucidum (MLS-SP), pineal gland shift (PGS), the ratio of the ipsilateral to contralateral midbrain width (IMW/CMW), and other exploratory markers of radiographic shift/compression. Pupil reactivity was measured using an automated infrared pupillometer (NeurOptics^®^, Inc.), specifically the proprietary algorithm for Neurological Pupil Index^®^ (NPi). We used rank-normalization and linear mixed-effects models, stratified by diagnosis and hemorrhagic conversion, to test associations of radiographic markers of shift and asymmetric pupil reactivity (Diff NPi), adjusting for age, lesion volume, Glasgow Coma Scale, and osmotic medications.

**Results:**

Of 53 patients with 74 CT images, 26 (49.1%) were female, and median age was 67 years. MLS-SP and PGS were greater in patients with IPH, compared to patients with ischemic stroke (6.2 v. 4.0 mm, 5.6 v. 3.4 mm, respectively). We found no significant associations between pupil reactivity and the radiographic markers of shift when adjusting for confounders. However, we found potentially relevant relationships between MLS-SP and Diff NPi in our IPH cohort (β = 0.11, SE 0.04, *P* = 0.01), and PGS and Diff NPi in the ischemic stroke cohort (β = 0.16, SE 0.09, *P* = 0.07).

**Conclusion:**

We found the relationship between midline shift and asymmetric pupil reactivity may differ between IPH and ischemic stroke. Our study may serve as necessary preliminary data to guide further prospective investigation into how clinical manifestations of radiographic midline shift differ by diagnosis and proximity to the midbrain.

## Introduction

One of the most feared early complications of acute ischemic stroke and intraparenchymal hemorrhage (**IPH**) is anatomic shift of midline structures (1). Mass effect on midline structures can cause secondary and life-threatening injury (1) and is therefore closely monitored clinically and radiographically. In critically ill patients with acute intracranial injury, mass effect is best visualized through serial radiographic imaging, particularly head Computed Tomography (**CT**) due to its speed and accessibility. Midline shift, often measured at the septum pellucidum, is a quantitative marker used to assess severity and track mass effect trajectory over time. However, even with CTs serial imaging is logistically limited by scanner and transport availability, timing, and radiation concerns (2, 3). Other modalities, including ultrasound, are dependent on adequate temporal windows, which are not present in a large percentage of patients.

Therefore, current clinical pathways rely on recognition of non-specific clinical signs of deterioration such as decreased arousal, which is also associated with fever, medications, or metabolic abnormalities (4, 5). More objective signs of impending herniation occur late, including ipsilateral or contralateral motor impairment, or subjective recognition of pronounced absence of pupil reactivity and large pupil diameter (size), traditionally referred to as “a fixed and dilated pupil”.

While pupil size and reactivity has historically been assessed qualitatively, the increasing use of automated infrared pupillometers in neurocritical care offers the opportunity to study the association of both evolving brain injury and evolving *quantitative* pupil characteristics, including constriction velocity, dilation velocity, latency, pupil size, and pupil reactivity *via* the Neurological Pupil Index^®^ (NPi) (6). While the use of the device is growing, few studies have rigorously examined the presence and effect sizes of quantitative pupil asymmetry or difference in NPi (**Diff NPi**) and multiple radiographic markers of midline shift or compression.

Understanding the relationship between asymmetric pupil changes and radiographic midline shift has potential high clinical utility. Pupil checks are frequent, non–invasive, and safe. If certain characteristics can identify the presence, relative location, and degree of mass effect, clinicians can more expeditiously and specifically order confirmational radiographic imaging to inform management and prognosis.

To address the need for non-invasive and reproducible bedside indicators of evolving intracranial injury, a better understanding of the relation between quantitative pupil characteristics and radiographic markers of midline shift and compression are needed. Other studies and case series suggest that there is an association between pupil reactivity and midline shift at the level of the septum pellucidum (7) and pineal gland (8). Because pupillary pathways traverse the midbrain, we hypothesized that differences in pupil reactivity would be significantly associated with compression of midline structures, including midline shift at the septum pellucidum (**MLS-SP**), pineal gland shift (**PGS**), and unilateral midbrain compression, defined as the ratio of the ipsilateral midbrain width to the contralateral midbrain width (**IMW/CMW**). A more comprehensive understanding of the relation between midline structure displacement and pupil characteristics could provide clinicians with non-invasive and accessible information to assist in localizing injury and guiding treatment decisions for evolving intracranial pathology.

## Materials and methods

We conducted a four-center retrospective cohort study of patients with large acute supratentorial ischemic stroke or primary IPH from the Brigham and Women's Hospital (**BWH**), Massachusetts General Hospital (**MGH**), Boston Medical Center (**BMC**), and University of Texas Southwestern Medical Center (**UTSW**) Neurointensive Care Units admitted between 2016 and 2020. Study participants were selected as a convenience sample of adult patients with radiographic evidence of stroke larger than one third of the Middle Cerebral Artery (**MCA**) or IPH volume >30 mm^3^ on head CT performed within 75 min of quantitative pupillometry. Seventy-five min was chosen because it provided the best opportunity to obtain quantitatively collected pupil measurements at a close time-period to either emergent or scheduled imaging. CT images are typically obtained by treating clinicians at their discretion. Typical reasons include neurologic decline (including anisocoria), or surveillance imaging in the absence of neurologic decline. At these institutions quantitative pupillometry is standard practice at 2-h (MGB), and at the time of any neurological checks which includes every one, two, or 4 h depending on the frequency of neuro checks ordered (BMC and UTSW). Images were excluded from the analysis if they had significant acute contralateral or posterior fossa injury (> 1/3 any vascular territory), had incomplete pupil measurements (missing right or left pupil observations), or were performed after surgical decompression, external ventricular drain placement, or had greater than trace intraventricular hemorrhage.

### Data collection

We collected demographic, procedural, Glasgow Coma Scale (**GCS**) (9), and osmotic medications [including mannitol or hypertonic saline (23.4 or 3%)] from the electronic medical record at home institutions. Quantitative pupillometry data were recorded and collected using the NeurOptics NPi-200 (NeurOptics Inc.,) pupillometer. Trained nursing staff conducted quantitative pupillometry as standard of care every 1–2 h in participating neurocritical care units. In addition to recording pupil size and constriction velocity, the pupillometer calculates the Neurological Pupil index (**NPi**), an algorithm that uses resting and constricted pupil size, percent change, constriction and dilation velocity, and latency (10, 11). NPi values range between 0 and 5. Values < 3 are considered “abnormal” and values ≥3 normal based on studies of normal populations. Its relation to constriction velocity has been externally examined in prior studies (11). There is no standard practice for administering osmotic medication or image collection following abnormal NPis alone at these institutions.

**Figure 1:**
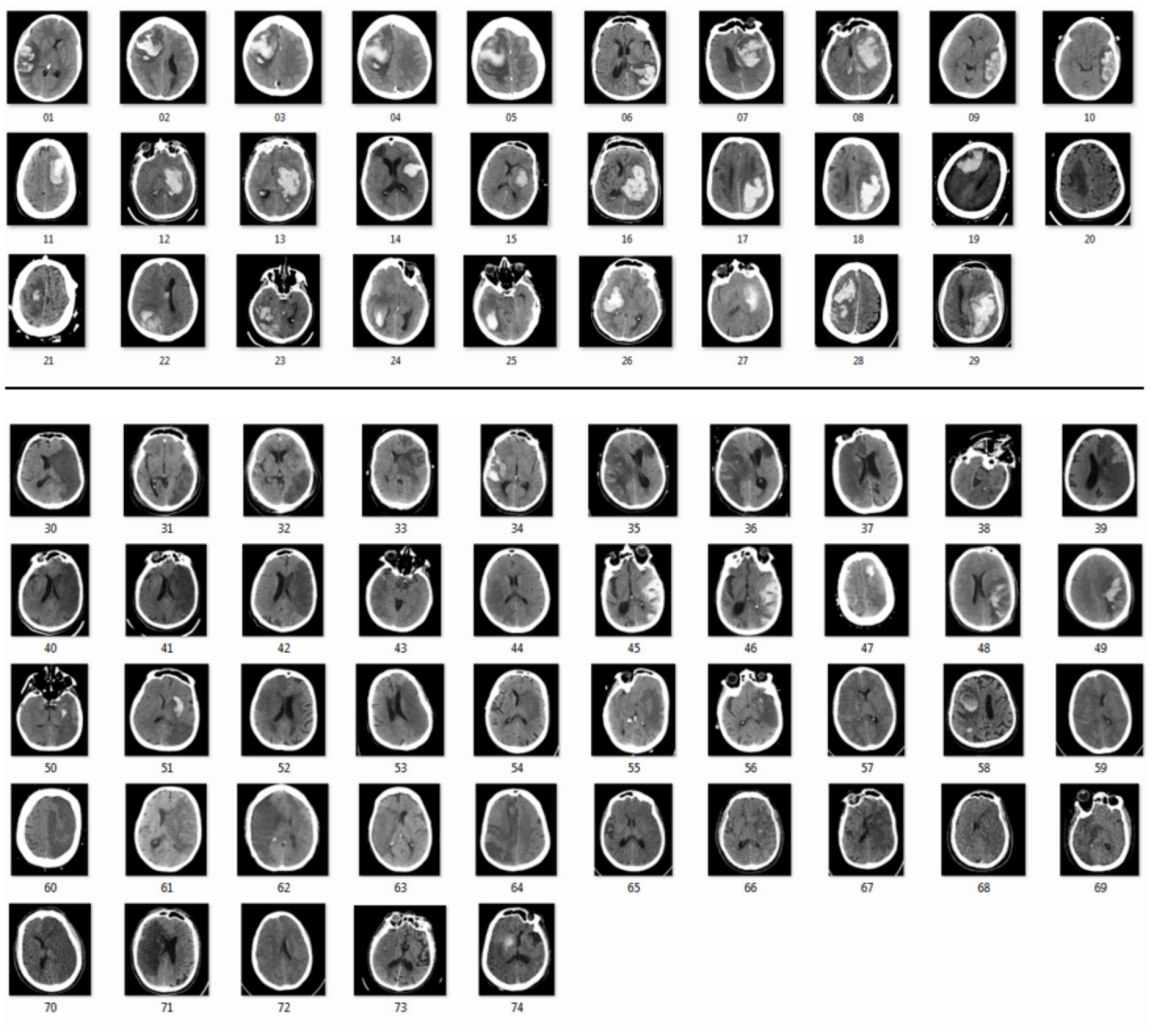
Head computed tomography of intraparenchymal hemorrhage patients (01–29) and ischemic stroke patients (30–74) from four different sites from 2016 to 2020.

Digital Imaging and Communications in Medicine (DICOM) head CT images were obtained from each of the four collaborating centers and were stored in a centralized database (Figure 1). A trained M.D. (FB) standardized head CT images from eligible study participants using semi-automatic registration on AnalyzePro14.0 using the sagittal view as the reference image (Supplementary Figure 1). MLS-SP was measured at the level of the Septum Pellucidum at its maximal deviation from the midline (12). PGS was measured at the level where the pineal gland at its point of maximal deviation as it had the best inter-rater reliability for measurement purposes. Moreover, authors felt that any bias introduced by measuring at the maximal deviation would most likely err toward the null, as some pineal calcifications are significantly larger at baseline than others and less likely result in a spurious positive association. To measure IMW, a straight line was drawn connecting the ipsilateral side of the cerebral peduncle (nearest to the associated temporal lobe) to the midbrain midline defined as the perpendicular line connecting the interpeduncular cistern with the quadrigeminal cistern. CMW was measured by connecting the midbrain midline to the contralateral cerebral peduncle, and the ratio between the two (**IMW/CMW**) was subsequently calculated. Information on other radiographic markers of midline shift and compression are included in the Supplementary Methods. A two-way intraclass correlation coefficient (**ICC**) was used to measure the reliability of ratings of radiographic markers that were measured by assigned investigators (IK, OB, and BP). Reliability was interpreted based on the 95% confident interval of the ICC values: < 0.5 (poor), 0.5–0.75 (moderate), 0.75–0.9 (good), and >0.9 (excellent).

The study was approved by our local Institutional Review Boards (H-37699, 2016P002718). The study was exempt from requiring consent because pupillometry assessment remains standard of care and no intervention was introduced to research participants. We prepared this report according to Strengthening the Reporting of Observational Studies in Epidemiology (**STROBE**) reporting guidelines.

### Variables

Our primary outcome was the continuous absolute difference in pupil reactivity between left and right eye (**Diff NPi**). A description and abbreviations of all quantitative pupil metrics collected and analyzed are included in Supplementary Table 1. Our three primary exposures included MLS-SP, PGS, and IMW/CMW. MLS-SP and PGS were chosen as these markers are frequently quantified and have prior evidence suggesting they are associated with pupillary changes. We selected IMW/CMW as our marker of intrinsic midbrain involvement as we postulated that a marker of midbrain compression would most affect asymmetric pupil reactivity and be less subject to natural variations in baseline configurations of the midbrain than midbrain shift. For our multivariable models, we selected hypothesized confounders that affect both pupil reactivity/size and midline shift based on the literature including age (13), lesion volume, arousal *via* the GCS score (14), and osmotic medications (15). Because other pupil influencing medications are not necessarily causally related to radiographic midline shift, these were not included as covariates. However, we have included a list of potentially pupil-influencing medications (Supplementary Table 2) prior to pupil observation in Supplementary Table 3.

Exploratory markers of midline shift and compression included Interpeduncular Shift (**IPS**)-measured on the axial slice in which the interpeduncular cistern was most visible, as a perpendicular line from the midline connecting the Crista Galli and the Posterior occipital protuberance), the ratio of Midbrain width to midbrain length (**MW/ML**—defined by the ratio of the width of the midbrain from cerebral peduncles over the length of the midbrain from the interpeduncular to intercollicular cistern), and the interpeduncular angle (**IA**—defined as the minimum measured angle between the cerebral peduncles). More information on our protocols are included in Supplementary Methods.

### Statistical analysis

Baseline characteristics are reported in Table 1, with median or proportions and interquartile range (**IQR**) as appropriate. We transformed our pupil variables using rank normalization to satisfy the normality assumption (16). To test the association between our primary outcome, the continuous difference in pupil reactivity (Diff NPi) and radiographic exposures, we first conducted univariate linear mixed effects models accounting for correlations between patients with multiple scans using a random effect. To assess collinearity between our radiographic markers of interest, we tested correlation based on the Spearman coefficient (17). We constructed a multivariable model testing the association of MLS-SP, PGS, and IMW/CMW adjusting for potential confounders. We used two-sided tests and a Benjamini-Hochberg correction with a False Detection Rate of 0.10 to assess significance for our hypotheses that markers of midline shift and compression including MLS-SP, PGS, and IMW/CMW are significantly associated with Diff NPi in our total cohort and ischemic and IPH subgroups (18).

**Table 1:** Baseline patient and observation characteristics.

**Patient characteristics**	**Total**	**Ischemic stroke**	**IPH**
	***N* = 53 (100%)**	***N* = 34 (64.2%)**	***N* = 19 (35.8%)**
**Demographics and outcomes**
Age	67.0 (62.0–77.0)	66.5 (62.0–79.0)	67.0 (63.0–71.0)
Female	26 (49.1%)	17 (50.0%)	9 (47.4%)
**Race**	
*White*	23 (43.4%)	14 (41.2%)	9 (47.4%)
*Black*	9 (17.0%)	6 (17.7%)	3 (15.8%)
*Asian*	5 (9.43%)	4 (11.8%)	1 (5.3%)
Unknown^*^	16 (30.2%)	10 (29.3%)	6 (31.6%)
**Ethnicity**	
*Hispanic*	9 (17.0%)	7 (20.6%)	2 (10.5%)
*Non-hispanic*	42 (79.3%)	26 (76.5%)	16 (84.2%)
*Unknown*	2 (3.7%)	1 (2.9%)	1 (5.3%)
**Medical Institution**	
*Massachusetts General*	23 (43.4%)	12 (35.3%)	11 (57.9%)
*Brigham Women's*	8 (15.1%)	4 (11.8%)	4 (21.1%)
*Boston Medical Center*	14 (26.4%)	12 (35.3%)	2 (10.5%)
*University of Texas Southwestern*	8 (15.1%)	6 (17.6%)	2 (10.5%)
**Death at discharge**	35 (47.3%)	23 (51.1%)	12 (41.4%)
**Observation characteristics**	**Total**	**Ischemic stroke**	**IPH**
	***M*** = **74 (100%)**	***M*** = **45 (60.8%)**	***M*** = **29 (39.2%)**
**Medical institutions**	
*Massachusetts General Hospital*	38 (40.5%)	20 (44.4%)	18 (62.1%)
*Brigham Women's Hospital*	14 (18.9%)	7 (15.6%)	7 (24.1%)
*Boston Medical Center*	14 (18.9%)	12 (26.7%)	2 (7.0%)
*University of Texas Southwestern*	8 (10.8%)	6 (13.3%)	2 (7.0%)
**Imaging characteristics**	
Lesion volume^**^ (mm^3^)	91.8 (53.1–183.0)	115.0 (60.6–248.8)	66.5 (47.0–135.0)
MLS-SP (mm)	4.9 (2.4–8.7)	4.0 (2.2–6.9)	6.2 (3.4–12.1)
PGS (mm)	4.1 (2.7–6.0)	3.4 (2.5–5.1)	5.6 (2.5–6.5)
**Quantitative pupil characteristics**	
Pupil Obs to head CT (min)	29.0 (21.0–47.8)	30.0 (22.0–50.0)	28.0 (21.0–45.0)
R/L NPi difference	0.2 (0.1–0.5)	0.2 (0.1–0.6)	0.2 (0.1–0.3)
R/L resting diff size (mm)	0.5 (0.2–0.7)	0.5 (0.3–0.7)	0.3 (0.2–0.5)
Average NPi	4.4 (3.7-4.8)	4.5 (3.4–4.8)	4.4 (3.9–4.8)
**Covariate characteristics**			
GCS within 60 min^I^	10.0 (7.7–12.0)	10.0 (7.0–13.0)	9.0 (8.0–12.0)
Osmotic Med within 60 min	13 (17.6%)	7 (15.6%)	6 (20.7%)

Medians (Q1-Q3) or N (%).

IPH, Intraparenchymal Hemorrhage; M, Number of head Computed Tomography images; N, Number of patients; NPi, Neurological Pupil Index; Obs, Observations; R/L, Right/Left.

^*^Unknown, Native American, or Hawaiian Pacific Islander.

^**^Average lesion volume was used if there were more than one Head CT for a given patient.

^I^For patients with multiple head CTs, we reported the average of Glasgow Coma Scale closest to imaging.

We also conducted exploratory analyses of the association of quantitative pupil characteristics and a comprehensive list of potential radiographic markers of midline shift using similar univariate linear mixed effects models. We used RStudio Version 1.3.959 [(19). RStudio: Integrated Development for R. RStudio, PBC] for statistical analyses. Further details are available in Supplementary Methods.

## Results

**Figure 2:**
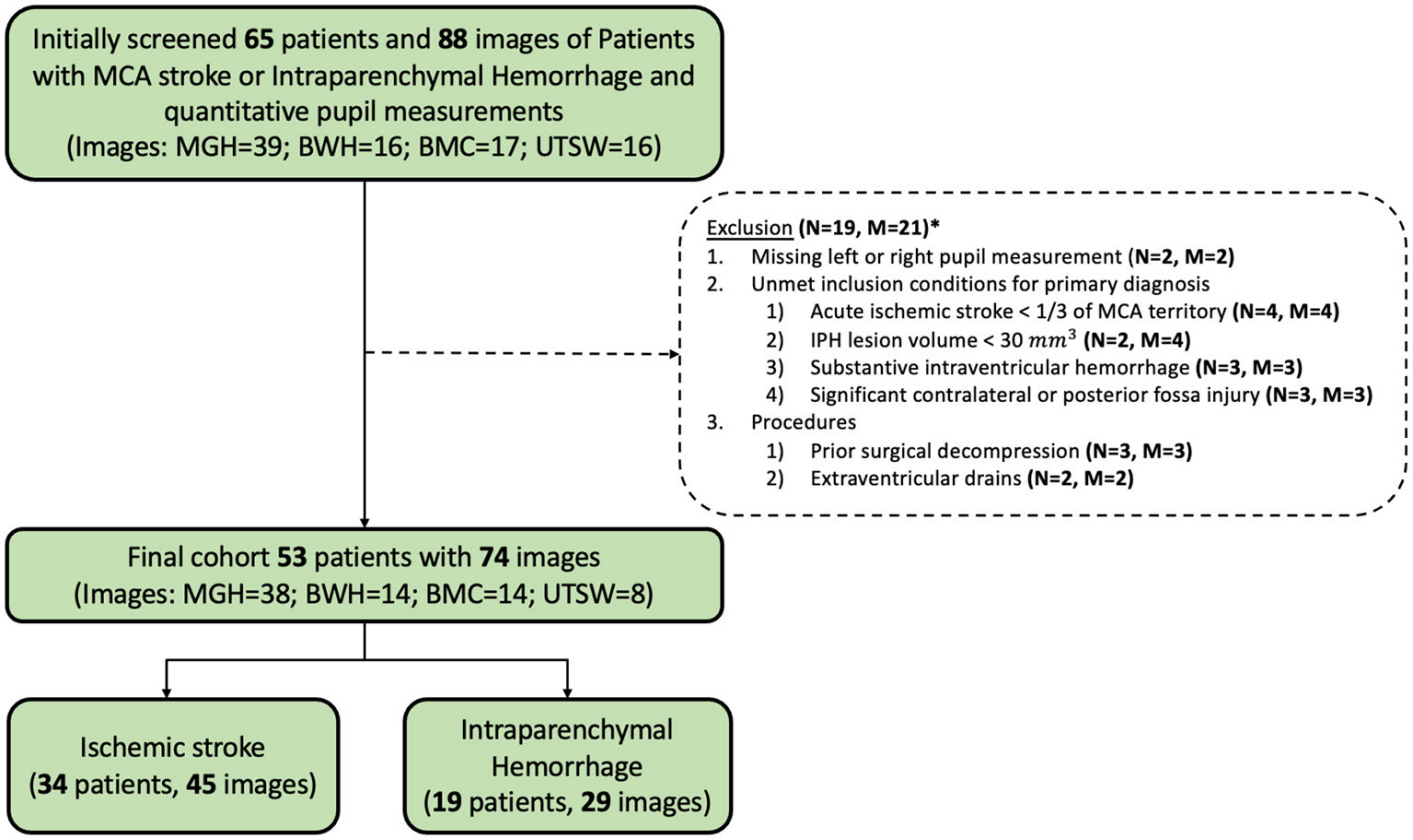
Inclusion and exclusion flow chart. M, Number of head CTs; N, Number of patients. *Some patients meet more than one exclusion criteria.

Fifty-three patients with 74 CT scans met our final eligibility criteria. Nineteen patients with 25 scans were excluded for < 1/3 MCA territory or < 30 cc IPH (*N* = 6), significant contralateral or posterior fossa brain injury (*N* = 3), substantive intraventricular hemorrhage (*N* = 3), external ventricular drains (*N* = 2), prior surgical decompression (*N* = 3), or missing left or right pupil measurements (*N* = 2; Figure 2). The final cohort consisted of patients with a median age of 67 (IQR: 62–77) years. Twenty-six (49.1%) patients were female, 8 were admitted to BWH, 23 to MGH, 14 to BMC, and 8 to UTSW. Nineteen patients (35.8%) had IPH and 34 patients (64.2%) had ischemic stroke (Table 1). Ten patients had 2 CTs, four had 3 CTs, and one had 4 CTs used for data analysis.

Of 74 CT scans, the median MLS-SP was 4.9 (2.4–8.7) mm, PGS was 4.1 (1.6–3.4) mm, and IMW/CMW was 0.93 (0.75–1.12). In our cohort, patients with IPH had larger shift and/or compression in midline structures as evidenced by MLS-SP (6.2 v. 4.0 mm), PGS (5.6 v. 3.4 mm), and IMW/CMW (0.92 v. 0.93). There was moderate correlation between the three primary radiographic outcomes (Spearman coefficient 0.11 for MLS-SP and IMW/CMW, 0.37 for PGS and IMW/CMW, and 0.58 for MLS-SP and PGS, respectively). The intercorrelation coefficients showed good reliability for MLS-SP (0.78; IQR: 0.58–0.88) and IMW/CMW (0.72; IQR: 0.33–0.94), and excellent reliability for PGS (0.90; IQR: 0.81–0.95).

Median Diff NPi among our 74 observations was 0.2 (IQR: 0.1–0.48), median Diff Size was 0.45 (IQR: 0.25, 0.65) mm, and median Min NPi was 4.3 (IQR: 3.4, 4.7). The ischemic stroke group had a median Diff NPi of 0.2, (IQR: 0.1, 0.6), median Diff Size of 0.65 mm (IQR: 0.3–0.74), and median Min NPi of 4.3 (IQR: 3.1–4.7), while the IPH group had a median Diff NPi of 0.2 (IQR: 0.1–0.3), median Diff Size of 0.5 mm (IQR: 0.18–0.5), and median Min NPi of 4.3 (IQR: 3.7–4.7). Median time between pupil observation and imaging was 29 min (IQR: 21–47.8; Table 1, Supplementary Table 3).

In our linear mixed effect univariate model accounting for intra-patient correlation, we found that PGS was associated with Diff NPi (*P* = 0.05) in the full patient cohort, and MLS-SP was associated with Diff NPi, Diff Size, and min NPi in the IPH Cohort (*p* < 0.01) (Table 2). A comprehensive list of our univarates associations is included in Supplementary Tables 4–7. After adjusting for confounders in our multivariable model, associations between Diff NPi and radiographic markers of midline shift did not meet the threshold for significance (Table 3) However, we found an intriguing relationship between Diff NPi and MLS-SP in our IPH subgroup, after adjusting for other markers of midline shift and midbrain compression (β = 0.11 increase in rank-normalized Diff NPi for every 1 mm increase in MLS-SP, SE 0.04, *P* = 0.01). Similarly, Diff Size was also significantly associated with MLS-SP in our IPH subgroup (β = 0.18 increase in rank-normalized Diff Size for every 1 mm increase in MLS-SP, SE 0.05, *P* < 0.01) (Supplementary Table 4). In our ischemic stroke cohort, PGS appeared to have the strongest relation with Diff NPi compared to other radiographic markers (β = 0.17 increase in rank-normalized Diff NPi for every 1 mm increase in PGS, SE 0.09, *P* = 0.07; Table 3). In a further subgroup analysis including only ischemic stroke scans with no hemorrhagic conversion, we also found a strong association between PGS and Diff NPi (β = 0.30, SE 0.12, *P* = 0.02; Supplementary Table 8).

**Table 2:** Univariate models accounting for inter-patient correlation.

	**MLS-SP**		**PGS Max**		**IMW/CMW**	
	**Beta (SE)**	***P***	**Beta (SE)**	***P***	**Beta (SE)**	***P***
**Full patient cohort (*****N*** **=** **53**, ***M*** **=** **74)**
Diff NPi	0.04 (0.03)	0.11	0.09 (0.05)	0.05	0.41 (0.30)	0.18
Diff size	0.04 (0.03)	0.16	0.02 (0.05)	0.62	0.30 (0.33)	0.37
Min NPi	−0.04 (0.03)	0.15	−0.07 (0.05)	0.12	−0.19 (0.31)	0.55
**Ischemic stroke cohort (*****N*** **=** **34**, ***M*** **=** **45)**
Diff NPi	0.00 (0.04)	0.93	0.13 (0.06)	0.06	0.65 (0.44)	0.14
Diff size	0.00 (0.04)	0.92	0.06 (0.06)	0.37	0.69 (0.40)	0.09
Min NPi	−0.01 (0.04)	0.75	−0.09 (0.07)	0.16	0.07 (0.45)	0.88
**Intraparenchymal hemorrhage cohort (*****N*** **=** **19**, ***M*** **=** **29)**
Diff NPi	0.09 (0.03)	< 0.01	0.11 (0.06)	0.11	0.19 (0.40)	0.63
Diff size	0.10 (0.04)	0.01	0.08 (0.08)	0.32	−0.07 (0.55)	0.90
Min NPi	−0.08 (0.03)	< 0.01	−0.09 (0.06)	0.14	−0.62 (0.42)	0.15

Diff NPi, Absolute difference in left and right Neurologic Pupil Index; Diff Size, Absolute difference in left and right resting pupil size; M, Number of head Computed Tomography images; Min NPi, Minimum NPi of the left and right eye; MLS, Midline Shift at septum pellucidum; N, Number of patients; NPi, Neurological Pupil index; PGS, Pineal Gland Shift; SE, Standard Error.

β coefficients are reported as an increase in one unit of transformed pupil outcome.

**Table 3:** Multivariable model assessing Diff NPi and radiographic markers of midline shift.

	**Beta (SE)**	***P***
**Full patient cohort (*****N*** **=** **53**, ***M*** **=** **74)**		
MLS-SP	0.01 (0.04)	0.69
PGS	0.07 (0.04)	0.24
IMW/CMW	0.65 (0.37)	0.08
Age	0.01 (0.01)	0.46
Lesion volume	0.00 (0.00)	0.04
GCS	−0.00 (0.05)	0.98
Osmotic medications	0.22 (0.31)	0.48
**Ischemic stroke cohort (*****N*** **=** **34**, ***M*** **=** **45)**		
MLS-SP	−0.05 (0.06)	0.40
PGS	0.17 (0.09)	0.07
IMW/CMW	0.84 (0.52)	0.12
Age	−0.00 (0.02)	0.96
Lesion volume	0.00 (0.00)	0.15
GCS	−0.00 (0.06)	0.99
Osmotic medications	0.20 (0.45)	0.66
**Intraparenchymal hemorrhage cohort (*****N*** **=** **19**, ***M*** **=** **29)**		
MLS-SP	0.11 (0.04)	0.01
PGS	−0.11 (0.08)	0.20
IMW/CMW	0.16 (0.47)	0.74
Age	0.03 (0.02)	0.10
Lesion volume	0.01 (0.00)	0.04
GCS	0.08 (0.07)	0.29
Osmotic medications	0.16 (0.36)	0.67

GCS, Glasgow Coma Scale; IMW/CMW, Ipsilateral Midbrain Width/Contralateral Midbrain Width; MLS-SP, Midline Shift at Septum Pellucidum; PGS, Pineal Gland Shift.

In our exploratory analyses, we assessed potential relationships between quantitative pupil values and radiographic markers of MLS (Supplementary Table 9). On further analysis of the association of NPi and MLS-SP in the IPH subgroup, we found that MLS-SP and contralateral NPi appeared to be related (β = −0.08, SE 0.03, *P* = 0.02). We also observed that in our full cohort, our ratio of IMW/CMW appeared to be significantly and negatively associated with contralateral resting pupil size (β = −0.88, SE 0.3, *P* < 0.01) and contralateral constriction velocity (β = −0.80, SE 0.29, *P* = 0.01) suggesting that as the ipsilateral width decreases in comparison to contralateral width, can affect the opposite pupil (Supplementary Tables 4–7).

## Discussion

Among the constellation of symptoms associated with herniation, asymmetric pupil reactivity and size are perhaps the most classic. Plum and Posner (20) expressed the view that in certain cases the diencephalon would not be the first structure compressed but that pupil signs might be an earlier feature than others in uncal herniation. Ropper and Shafran (21) found that the first indication of oculomotor (third) nerve compression is usually a sluggish or absent light reaction on the side of the mass, which may persist for hours or longer before the pupil actually enlarges.

Building on this background, modern studies have sought to confirm the relationship between quantitative measurement of pupil reactivity/size and evidence of mass effect. Mass effect is commonly clinically quantified by radiographic midline shift, and increased midline shift at various levels including the septum pellucidum and pineal gland have been associated with drowsiness, stupor, and pupil changes in seminal case series (8). In modern investigations, Osman et al. found significant associations in their study of 134 patients with MLS-SP and NPi (7). However, midline shift was associated with the ipsilateral pupil only in the rightward but not leftward direction.

In our study, we present preliminary data seeking further clarification of the relationship between radiographic shift/compression prior to herniation and asymmetric pupil reactivity, or the difference in NPi. Characterizing this relationship can help clinicians better interpret non-invasively measured physiologic characteristics that signify early neurological worsening in acutely ill patients. We hypothesized that like Osman, et al. there is a significant relationship between MLS-SP and difference in NPi. Further, we also hypothesized that there would be a significant association with PGS, as others have found that the pineal gland is more closely associated with diminished arousal. Finally, we explored whether there was a reliable relationship between other radiographic markers of midbrain compression and asymmetric pupil reactivity.

Unlike Osman, et al. (7) we did not find a significant association between markers of radiographic shift and Diff NPi when adjusting for other confounders including age, lesion volume, GCS, and osmotic medications. However, MLS-SP appeared to have a stronger association with asymmetric pupil reactivity in IPH patients than in ischemic stroke patients. We also found that PGS, while also not meeting our predetermined threshold for statistical significance, appeared more strongly associated with Diff NPi in our ischemic stroke cohort than in patients with IPH. PGS continued to appear more strongly associated with Diff NPi in patients with no hemorrhagic transformation. Our findings raise the question of whether the clinical significance and manifestations of radiographic midline shift at different anatomic levels varies by diagnosis and intracerebral hemorrhage.

One possible hypothesis for why MLS-SP might have a stronger association with IPH may be because of more substantial disruption of cortical pathways responsible for attention that modulate pupil reactivity and size. A recent study showed that pupillary responses and differences in size can be elicited when no differential light is presented nor expected, when participants were asked to recall dark or bright objects, suggesting that attention can play a role in pupil modulation independent of external visual sources (22). While there is no literature that we are aware of studying IPH location and pupil dysfunction, Peinkhofer, et al. (23) evaluated the pupillary light reflex in 25 ischemic stroke patients with insular cortex or prefrontal eye field involvement and compared them to controls. They failed to find a correlation between the pupillary light reflex in the prefrontal eye field or insula (23). If patients with ischemic stroke have less disruption of cortical pupil-modulating pathways compared to patients with IPH when controlling for size, it may be a reason why we observed that MLS-SP is less strongly associated with asymmetric pupil reactivity in the ischemic stroke group.

PGS on the other hand, has been shown in qualitative case series to be strongly associated with decreased level of consciousness, which often but not always accompanies asymmetric change in pupil reactivity (8). However, similar to the discrepancy between the strength of the association of asymmetric pupil reactivity and MLS at the septum pellucidum, we also found that the extent of the association between PGS and Diff NPi also differed between ischemic stroke and IPH cohorts. One possibility for our observation may be related to the dynamic nature of pupillary change.

In our prior work on anisocoria, we found that up to 63% of patients experienced new onset anisocoria (pupil size difference of ≥1 mm) at least once during their hospitalization, and that it occurred up to 10% of all pupillary measurements (24). However, after new onset anisocoria occurred, it was often transient. Of the 45 MGH and BMC patients, 28 of the 29 patients who had a Diff NPi of >0.7 (considered abnormal by the manufacturer), normalized within an average of 2.7 h. Whether these values normalized in response to targeted therapy was out of scope for the present study, but these observations demonstrate that pupil characteristics can fluctuate between abnormal and normal values. Others posited that fluctuations in pupil size and shape may be a transtentorial sign before frank pupillary enlargement, and also transient features during recovery of light reaction may represent incomplete stages of compression of the pupilloconstrictor fibers of the third nerve (25). One possible reason for why PGS and Diff NPi did not appear to strongly correlate in the IPH cohort compared to the ischemic stroke cohort may be because Diff NPi may be optimally detected at the time of the more rostral increase in MLS-SP (which typically occurs prior to PGS) and potentially normalized by the time PGS occurred. While the clinical significance of fluctuating pupil abnormalities is still unclear, foundational work including our methodological and preliminary examination of the relation between radiographic shift and pupil observations close in time is a necessary step toward a better understanding of how to interpret quantitative pupil characteristics.

We did not find meaningful associations between radiographic markers of midbrain compression and pupil reactivity. One reason for why we may not have observed significant associations for some of our markers of midbrain compression include variation in the size and configuration of tentorial openings. Because of wide ranges in the width of the incisura (2–4 cm), and from the edge of the midbrain to the tentorial margin (0–6.6 mm) (26), age-related atrophy may play a role in changing the outline of the opening between patients (27, 28), and almost certainly plays a role in how mass effect shifts rather than compresses neuronal pathways.

Our primary marker, IMW/CMW, appears to have an inverse relationship with both contralateral NPi and constriction velocity (Supplementary Table 3). While it has been reported that initial pupil involvement contralateral to the injury has been seen in up to 10% of pupillary changes due to subdural hematoma (29), there has been no unifying theory to adequately explain its occurrence. Some suggest that contralateral pupil impairment may be due to uncal herniation on the opposite side, while others believe this is almost impossible once the midbrain has shifted over and perimesencephalic cisterns are closed off (25). An alternative mechanism is that the posterior cerebral artery on the side of the mass may be higher than its opposite (resulting in less stretching of the ipsilateral third nerve in comparison to the contralateral side). Ultimately, we agree that different configurations are likely responsible for pupil enlargement (ipsilateral or contralateral) in individual cases due to difference in the shape and size of tentorial openings and in the course of the third nerves (25). Our findings suggest that as the ipsilateral midbrain width decreases in comparison to the contralateral midbrain width, subtle contralateral pupil changes may occur. Whether our observations are simply a product of multiple tests of association, or hints at irritation of the contralaterally running tract of the afferent pupillary light reflex pathway at the level of the midbrain is unclear. Further work would be needed to clarify the association between quantitative contralateral pupil response to acute injury locations.

### Limitations and strengths

We acknowledge several limitations to our study. Our sample size is small, limiting how definitively we could assess potentially significant associations. Moreover, in the measurement of our radiographic markers of interest, differences in imaging angle could impact how data was collected within one axial plane. We also did not have a baseline scan on each patient to use as a comparison. We attempted to mitigate these limitations by a comprehensive protocol for normalization and measurement and included patients who had multiple scans that met criteria adjusting for correlation using linear mixed effects regression. However, though interrater reliability of MLS-SP and PGS was good to excellent, we acknowledge that inter-rater reliability, especially of IMW/CMW, may have limited our ability to accurately observe some relationships. We conducted multiple tests of association, increasing the potential for false positive findings. We attempted to allay the limitation by selecting three primary hypotheses and an appropriate statistical correction. The remainder of our analyses are hypothesis-generating for more definitive studies. Given the transformations that were required to normalize our data, we caution any interpretations concerning effect size. Because our study was observational and retrospective, we cannot exclude residual confounding or establish causal relations. We did not have information on cognitive load, pain, or ambient light levels, which have been reported to affect pupil characteristics (14). We were unable to adjust for all potential residual confounders including potential pupil influencing medications (15, 30), which can affect pupil size and reactivity. However, we have included data that occurred prior to pupil measurments and prior to imaging in Supplementary Table 3.

Despite limitations, our study has the following strengths. We conducted a four-center study with pupil observations conducted within 75 min of a radiographic image to assess the relation between quantitative pupil markers and radiographic markers of midline shift. Other work in this area has previously used larger time windows (6 h) (7), which limits the study of the associations between immediate pupillary reaction in response to mass effect. We comprehensively measured both established and novel markers of shift and compression. We adjusted for patient- and pupil-level covariates including radiographic markers of MLS and compression at various anatomic levels, demographics, osmotic medications, and arousal state. We believe that our results can provide important information for further prospective studies.

## Conclusion

Our work describes the methodology and potential associations between radiographic markers of midline shift and quantitative pupil characteristics. The results suggest that the relation between midline shift at various levels and asymmetric pupil reactivity may differ for different diagnoses. Our study may serve as necessary preliminary data to guide further prospective investigation into how clinical manifestations of radiographic midline shift differ by diagnosis and proximity to the mid.

## Data availability statement

Due to protected health information, all data will be made available upon request after appropriate data use agreements with the participating institutions.

## Ethics statement

The studies involving human participants were reviewed and approved by Partners Human Research Committee; Harvard Medical Center Integrated Network for Subject Protection In Research; Boston University. Written informed consent for participation was not required for this study in accordance with the national legislation and the institutional requirements.

## Author contributions

SMS, DO, and CO conceived and designed the overall study. IK, OB, BP, HS, KS, SES, NS, VA, and BL organized and administered collected data for modeling. AM reviewed interrater reliability. IK, BP, and CO performed statistical analysis. JD reviewed analysis and interpretation. IK, OB, BP, and CO wrote the manuscript. HS, DO, KS, SES, NS, VA, BL, SMS, JD, AM, DG, and CO helped with reviews and revision. CO provided overall study direction and critical review. All authors contributed to the article and approved the submitted version.

## Funding

This study was funded by National Institute of Neurological Disorders and Stroke (NINDS) Research Program Award (NIH/NINDS K23NS116033).

## Conflict of interest

The authors declare that the research was conducted in the absence of any commercial or financial relationships that could be construed as a potential conflict of interest.

## Publisher's note

All claims expressed in this article are solely those of the authors and do not necessarily represent those of their affiliated organizations, or those of the publisher, the editors and the reviewers. Any product that may be evaluated in this article, or claim that may be made by its manufacturer, is not guaranteed or endorsed by the publisher.

## Abbreviations

CT: Computed Tomography
Diff Npi: Difference in Npi
GCS: Glasgow Coma Scale
IA: Interpeduncular Angle
IMW/CMW: Ratio of The Ipsilateral Midbrain Width/Contralateral Midbrain Width
IPH: Intraparenchymal Hemorrhage
IPS: Interpeduncular Shift
MCA: Middle Cerebral Artery
MLS-SP: Midline Shift at The Septum Pellucidum
MW/ML: Ratio of Midbrain Width to Midbrain Length
NPi: Neurological Pupil Index
PGS: Pineal Gland Shift.
