# Supplementary material for: Quantitative pupillometry and radiographic markers of intracranial midline shift: A pilot study
Source: Front Neurol. 2022 Dec 6;13:1046548. doi: 10.3389/fneur.2022.1046548 (PMC9763295; doi:10.3389/fneur.2022.1046548)
Supplement: Supplementary file 1 [file Table_1.docx]

| **Pupil Characteristics** | **Definition** |
| --- | --- |
| Diff NPi | Difference in Neurological Pupil index (NPi) between left and right eyes |
| Diff Size | Difference in resting pupil size between left and right eyes |
| iSize | Resting pupil size of the eye on the ipsilateral side of stroke or IPH |
| cSize | Resting pupil size of the eye on the contralateral side of stroke or IPH |
| Min NPi | Numerical value of the pupil with the lower NPi |
| iNPi | NPi of the eye on the ipsilateral side of stroke or IPH |
| cNPi | NPi of the eye on the contralateral side of stroke or IPH |
| Avg NPi | Average NPi between left and right eye |
| Avg Size | Average pupil size between left and right eye |
| Min CV | Minimum Constriction Velocity between left and right eye |
| iCV | Constriction velocity of the eye on the ipsilateral side of stroke or IPH |
| cCV | Constriction velocity of the eye on the contralateral side of stroke or IPH |
| Min DV | Minimum Dilation Velocity between left and Right Eye |
| Max Latency | Maximum Latency between left and right eye |

**Supplementary Table 1**. Description of Pupil Metrics
