# Supplementary material for: Quantitative pupillometry and radiographic markers of intracranial midline shift: A pilot study
Source: Front Neurol. 2022 Dec 6;13:1046548. doi: 10.3389/fneur.2022.1046548 (PMC9763295; doi:10.3389/fneur.2022.1046548)
Supplement: Supplementary file 2 [file Table_2.docx]

**Supplementary Table 2.** Potentially Pupil-Influencing Medications

| **Medication Category** | **Route** |
| --- | --- |
| **Osmotic Medications** | |
| Mannitol | IM/IV |
| Hypertonic Saline 23% | IM/IV |
| Hypertonic Saline 3% | IV |
| **Analgesic Medications** | |
| Acetaminophen | PO/IV |
| Codeine | PO |
| Gabapentin | PO |
| Hydrocodone | PO |
| Hydromorphone | IM/IV |
| Ibuprofen | PO |
| Methadone | PO |
| Morphine | PO/IM/IV |
| Oxycodone | PO |
| Tramadol | PO |
| Meperidine | IV |
| **Sedatives** | |
| Dexmedetomidine | IV |
| Fentanyl | IM/IV/Patch |
| Ketamine | IM/IV |
| Midazolam | IM/IV |
| Pentobarbital | IM/IV |
| Propofol | IM/IV |
| Alprazolam | PO |
| Clobazam | PO |
| Clonazepam | PO |
| Diazepam | PO/IM |
| Etomidate | IM/IV |
| Hydroxyzine | PO |
| Lorazepam | PO/IM/IV |
| Phenobarbital | PO/IM/IV |
| Trazodone | PO |
| **Vasopressors** | |
| Dopamine | IV |
| Epinephrine | IM/IV |
| Norepinephrine | IM/IV |
| Phenylephrine | IM/IV |
| Vasopressin | IM/IV |
| **Antihypertensive Medications** | |
| Nicardipine | IV |
| Amlodipine | PO |
| Atenolol | PO |
| Bumetanide | IV/IM |
| Captopril | PO |
| Carvedilol | PO |
| Chlorthalidone | PO |
| Clonidine | PO |
| Diltiazem | PO/IV |
| Dobutamine | IV |
| Doxazosin | PO |
| Esmolol | IV |
| Furosemide | PO/IM/IV |
| Hydralazine | PO/IM/IV |
| Hydrochlorothiazide | PO |
| Isosorbide Dinitrate | PO |
| Labetalol | PO/IM/IV |
| Lisinopril | PO |
| Losartan | PO |
| Metoprolol | PO/IM |
| Nifedipine | PO |
| Nimodipine | PO |
| Prazosin | PO |
| Propranolol | PO |
| Spironolactone | IM |
| **Stimulant Medications** | |
| Amantadine | PO |
| Methylphenidate | PO |
| Modafinil | PO |
| Anticholinergics |  |
| Albuterol | Nebulizer |
| Cyclobenzaprine | PO |
| Dantrolene | PO |
| Fluticasone Salmeterol | Nebulizer |
| Ipratropium Albuterol | Nebulizer |
| Ipratropium Bromide | Nebulizer |
| Prochlorperazine Edisylate | IM/IV |
| Scopolamine | Patch |
| Tiotropium | PO |
| Haloperidol Lactate | IM |
| Chlorpromazine | PO/IM/IV |
| Methocarbamol | PO |
| Quetiapine | PO |
| Atropine | PO/IV |
| Diphenhydramine | PO/IM/IV |
| Oxybutynin | PO |
| **Other medications** | |
| Budesonide | Nebulizer |
| Buspirone | PO |
| Cisatracurium | IV |
| Rocuronium | IV |
| Dextran | Ophthalmic drop |
| Digoxin | PO/IV |
| Hydroxypropyl | Ophthalmic drop |
| Latanoprost | Ophthalmic drop |
| Loratadine | PO |
| Montelukast | PO |
| Naloxone | PO |
| Polyvinyl Alcohol | Ophthalmic drop |
| Racepinephrine | Nebulizer |
| Tamsulosin | Ophthalmic drop |
| Timolol | Ophthalmic drop/Nebulizer |
| White petrolatum-mineral oil | Ophthalmic drop |
| Abb.: IV-Intravenous; IM-Intramuscular; PO-Oral | |
