# Supplementary material for: Quantitative pupillometry and radiographic markers of intracranial midline shift: A pilot study
Source: Front Neurol. 2022 Dec 6;13:1046548. doi: 10.3389/fneur.2022.1046548 (PMC9763295; doi:10.3389/fneur.2022.1046548)
Supplement: Supplementary file 3 [file Table_3.docx]

**Supplementary Table 3.** Patient Level Data (M=74)

| **Study ID** | **Site** | **Diagnosis** | **Time Difference Between Pupil Observation and Head CT (Min)** | **Pupil Influencing Meds**  **up to 60 Min Prior to**  **Pupil Observation** | **Osmotic Meds up to 60 Min Prior to**  **Head CT** | **tPA** | **MT** | **TICI Score** |
| --- | --- | --- | --- | --- | --- | --- | --- | --- |
| 1 | A | IPH | 17.0 | Mannitol |  |  |  |  |
| 3 | A | Ischemic Stroke | 65.0 | Hypertonic Saline 23% |  |  |  |  |
| 12 | A | Ischemic Stroke | 17.0 | Mannitol |  |  |  |  |
| 15 | B | IPH | 23.0 | Nicardipine |  |  |  |  |
| 16 | B | IPH | 55.0 | Mannitol | Yes |  |  |  |
| 17 | B | IPH | 34.0 | Acetaminophen |  |  |  |  |
| 24 | B | IPH | 55.0 | Labetalol |  |  |  |  |
| 29 | B | Ischemic Stroke | 12.0 |  | Yes |  |  |  |
| 32 | B | IPH | 26.0 | Mannitol |  |  |  |  |
| 47 | B | Ischemic Stroke | 30.0 |  | Yes |  |  |  |
| 51 | B | IPH | 9.0 | Hypertonic Saline 23% |  |  |  |  |
| 53 | C | Ischemic Stroke | 51.0 | Labetalol  Etomidate  Phenylephrine  Rocuronium |  | Yes | Yes | 3 |
| 54 | C | IPH | 13.0 | Nicardipine  Hypertonic Saline 3% |  |  |  |  |
| 55 | C | IPH | 28.0 | Phenylephrine  Propofol  Vasopressin |  |  |  |  |
| 56 | C | Ischemic Stroke | 26.0 | Acetaminophen |  | Yes |  |  |
| 57 | C | Ischemic Stroke | 16.0 | Norepinephrine |  |  |  |  |
| 58 | C | Ischemic Stroke | 30.0 | Mannitol |  |  |  |  |
| 59 | C | Ischemic Stroke | 19.0 | Acetaminophen |  |  |  |  |
| 60 | C | Ischemic Stroke | 29.0 | Nicardipine |  | Yes | Yes | 2a |
| 61 | D | Ischemic Stroke | 15.0 |  |  |  | Yes | 3 |
| 63 | D | IPH | 34.0 |  |  | Yes |  |  |
| 66 | D | Ischemic Stroke | 19.0 | Mannitol  Hypertonic Saline 3% |  |  | Yes | 0 |
| 67 | D | Ischemic Stroke | 42.0 | Metoprolol |  | Yes | Yes | 2c |
| 68 | D | Ischemic Stroke | 46.0 |  |  |  | Yes | 2b |
| 69 | D | Ischemic Stroke | 41.0 | Fentanyl |  |  |  |  |
| 70 | D | Ischemic Stroke | 52.0 | Mannitol |  |  | Yes | 2c |
| 71 | D | Ischemic Stroke | 27.0 | Mannitol  Fentanyl |  |  |  |  |
| 72 | D | Ischemic Stroke | 48.0 | Tramadol |  |  |  |  |
| 73 | D | Ischemic Stroke | 45.0 |  |  |  | Yes | 3 |
| 74 | D | Ischemic Stroke | 20.0 |  |  | Yes | Yes | 3 |
| Patients who did not receive any pupil influencing medications, osmotic medications, or interventions not included (M=44). Site includes Boston Medical Center, Brigham Women’s Hospital, Massachusetts General Hospital, and University of Texas Southwestern Medical Center; Abb.: M-Number of head Computed Tomography images; IPH-Intraparenchymal Hemorrhage; CT-Computed Tomography; tPA-Tissue plasminogen activator; MT-Mechanical Thrombectomy; TICI-Thrombolysis in Cerebral Infarction | | | | | | | | |
