# Supplementary material for: Quantitative pupillometry and radiographic markers of intracranial midline shift: A pilot study
Source: Front Neurol. 2022 Dec 6;13:1046548. doi: 10.3389/fneur.2022.1046548 (PMC9763295; doi:10.3389/fneur.2022.1046548)
Supplement: Supplementary file 4 [file Table_4.docx]

**Supplementary Table 4**. Unadjusted Models Accounting for Inter-Patient Correlation

|  | **MLS-SP** | | **PGS** | | **Max IPS** | |
| --- | --- | --- | --- | --- | --- | --- |
| **Full Patient Cohort (N = 53, M = 74)** | | | | | | |
|  | Beta (SE) | p | Beta (SE) | p | Beta (SE) | p |
| Diff NPi | 0.04 (0.03) | 0.11 | 0.09 (0.05) | 0.05 | 0.01 (0.06) | 0.99 |
| Diff Size | 0.04 (0.03) | 0.16 | 0.02 (0.05) | 0.62 | -0.00 (0.07) | 0.98 |
| iSize | -0.00 (0.03) | 0.92 | -0.04 (0.05) | 0.48 | -0.04 (0.07) | 0.52 |
| cSize | 0.02 (0.03) | 0.51 | -0.01 (0.05) | 0.91 | 0.03 (0.07) | 0.70 |
| Min NPi | -0.04 (0.03) | 0.15 | -0.07 (0.05) | 0.12 | -0.06 (0.06) | 0.40 |
| iNPi | -0.01 (0.03) | 0.69 | -0.02 (0.05) | 0.66 | -0.02 (0.06) | 0.77 |
| cNPi | -0.05 (0.03) | 0.08 | -0.07 (0.05) | 0.13 | -0.10 (0.06) | 0.12 |
| Avg NPi | -0.03 (0.03) | 0.30 | -0.05 (0.05) | 0.31 | -0.05 (0.06) | 0.44 |
| Avg Size | 0.01 (0.03) | 0.84 | -0.02 (0.05) | 0.70 | -0.03 (0.06) | 0.62 |
| Min CV | -0.05 (0.03) | **0.05** | -0.10 (0.05) | 0.04 | -0.07 (0.07) | 0.31 |
| iCV | -0.03 (0.03) | 0.35 | -0.09 (0.05) | 0.07 | -0.02 (0.07) | 0.79 |
| cCV | -0.06 (0.03) | **0.04** | -0.09 (0.05) | 0.06 | -0.02 (0.07) | 0.09 |
| Min DV | -0.03 (0.03) | 0.30 | -0.05 (0.05) | 0.27 | -0.04 (0.06) | 0.53 |
| Max Latency | 0.05 (0.03) | **0.05** | 0.06 (0.05) | 0.21 | 0.11 (0.06) | 0.09 |
| **Ischemic Stroke Cohort (N = 34, M = 45)** | | | | | | |
|  | Beta (SE) | p | Beta (SE) | p | Beta (SE) | p |
| Diff NPi | 0.00 (0.04) | 0.93 | 0.13 (0.06) | 0.06 | -0.01 (0.09) | 0.90 |
| Diff Size | 0.00 (0.04) | 0.92 | 0.06 (0.06) | 0.37 | -0.08 (0.09) | 0.37 |
| iSize | 0.01 (0.04) | 0.77 | -0.00 (0.06) | 1.00 | -0.02 (0.08) | 0.80 |
| cSize | 0.04 (0.04) | 0.31 | 0.02 (0.06) | 0.78 | 0.08 (0.08) | 0.34 |
| Min NPi | -0.01 (0.04) | 0.75 | -0.10 (0.07) | 0.16 | -0.04 (0.09) | 0.68 |
| iNPi | -0.00 (0.03) | 0.94 | -0.04 (0.06) | 0.50 | 0.01 (0.08) | 0.87 |
| cNPi | -0.03 (0.04) | 0.52 | -0.07 (0.07) | 0.31 | -0.10 (0.09) | 0.29 |
| Avg NPi | -0.01 (0.04) | 0.86 | -0.06 (0.07) | 0.34 | -0.03 (0.09) | 0.75 |
| Avg Size | 0.03 (0.04) | 0.42 | 0.01 (0.06) | 0.82 | 0.03 (0.08) | 0.69 |
| Min CV | -0.03 (0.04) | 0.45 | -0.12 (0.06) | **0.05** | -0.04 (0.08) | 0.60 |
| iCV | -0.02 (0.04) | 0.59 | -0.13 (0.06) | **0.04** | -0.05 (0.09) | 0.53 |
| cCV | -0.04 (0.03) | 0.24 | -0.08 (0.05) | 0.14 | -0.10 (0.06) | 0.13 |
| Min DV | -0.03 (0.04) | 0.52 | -0.08 (0.07) | 0.25 | -0.08 (0.09) | 0.38 |
| Max Latency | 0.06 (0.04) | 0.12 | 0.07 (0.06) | 0.23 | 0.07 (0.08) | 0.38 |
| **Intraparenchymal Hemorrhage Cohort (N = 19, M = 29)** | | | | | | |
|  | Beta (SE) | p | Beta (SE) | p | Beta (SE) | p |
| Diff NPi^*^ | 0.09 (0.03) | **<0.01** | 0.11 (0.06) | 0.11 | 0.02 (0.08) | 0.86 |
| Diff Size | 0.10 (0.04) | **0.01** | 0.08 (0.08) | 0.32 | 0.09 (0.10) | 0.40 |
| iSize | 0.01 (0.04) | 0.83 | -0.02 (0.09) | 0.80 | -0.08 (0.10) | 0.44 |
| cSize | 0.02 (0.04) | 0.67 | 0.01 (0.09) | 0.90 | -0.03 (0.11) | 0.80 |
| Min NPi | -0.08 (0.03) | **0.01** | -0.09 (0.06) | 0.14 | -0.05 (0.08) | 0.51 |
| iNPi | -0.04 (0.03) | 0.26 | -0.03 (0.07) | 0.67 | -0.03 (0.08) | 0.76 |
| cNPi | -0.08 (0.03) | **0.02** | -0.11 (0.07) | 0.12 | -0.08 (0.08) | 0.35 |
| Avg NPi | -0.07 (0.03) | **0.02** | -0.08 (0.06) | 0.22 | -0.05 (0.08) | 0.51 |
| Avg Size | -0.00 (0.04) | 0.93 | -0.01 (0.09) | 0.90 | -0.08 (0.10) | 0.42 |
| Min CV | -0.08 (0.04) | **0.05** | -0.09 (0.09) | 0.34 | -0.10 (0.11) | 0.36 |
| iCV | -0.02 (0.04) | 0.56 | -0.03 (0.86) | 0.72 | 0.01 (0.10) | 0.96 |
| cCV | -0.08 (0.05) | 0.09 | -0.12 (0.10) | 0.24 | -0.17 (0.12) | 0.18 |
| Min DV | -0.05 (0.03) | 0.08 | -0.05 (0.06) | 0.39 | -0.00 (0.08) | 1.00 |
| Max Latency | 0.08 (0.04) | 0.07 | 0.12 (0.09) | 0.20 | 0.18 (0.10) | 0.09 |
| Abb.: Diff NPi-Absolute difference in left and right Neurologic Pupil Index; Diff Size-Absolute difference in left and right resting pupil size; IMW/CMW-Ipsilateral Midbrain Width/Contralateral Midbrain Width; M-Number of head Computed Tomography images; Max IPS-Maximum Interpeduncular Shift; Min NPi-Minimum NPi of the left and right eye; MLS-SP-Midline Shift at Septum Pellucidum; N-Number of patients; NPi-Neurological Pupil index; PGS-Pineal Gland Shift; SE-Standard Error. $\beta$ coefficients are reported as an increase in one unit of transformed pupil outcome using rank normalization. | | | | | | |
