# Supplementary material for: Quantitative pupillometry and radiographic markers of intracranial midline shift: A pilot study
Source: Front Neurol. 2022 Dec 6;13:1046548. doi: 10.3389/fneur.2022.1046548 (PMC9763295; doi:10.3389/fneur.2022.1046548)
Supplement: Supplementary file 5 [file Table_5.docx]

**Supplementary Table 5**. Unadjusted Models Accounting for Inter-Patient Correlation (cont)

|  | **IMW** | | **CMW** | | | **IMW/CMW** | |
| --- | --- | --- | --- | --- | --- | --- | --- |
| **Full Patient Cohort (N = 53, M = 74)** | | | | | | | |
|  | Beta (SE) | p | Beta (SE) | | p | Beta (SE) | p |
| Diff NPi | -0.02 (0.04) | 0.59 | 0.00 (0.04) | | 0.94 | 0.41 (0.30) | 0.18 |
| Diff Size | 0.00 (0.04) | 1.00 | 0.00 (0.05) | | 0.92 | 0.20 (0.33) | 0.37 |
| iSize | -0.02 (0.03) | 0.58 | -0.04 (0.04) | | 0.35 | -0.30 (0.30) | 0.31 |
| cSize | -0.04 (0.04) | 0.24 | -0.02 (0.05) | | 0.65 | -0.88 (0.30) | **<0.001** |
| Min NPi | -0.04 (0.04) | 0.29 | 0.04 (0.04) | | 0.40 | -0.19 (0.31) | 0.55 |
| iNPi | -0.07 (0.04) | 0.08 | 0.03 (0.04) | | 0.54 | -0.16 (0.32) | 0.62 |
| cNPi | -0.03 (0.04) | 0.43 | 0.06 (0.04) | | 0.15 | -0.07 (0.32) | 0.82 |
| Avg NPi | -0.05 (0.04) | 0.20 | 0.05 (0.04) | | 0.31 | -0.10 (0.31) | 0.75 |
| Avg Size | -0.06 (0.03) | 0.04 | -0.03 (0.04) | | 0.54 | -0.73 (0.26) | **0.01** |
| Min CV | -0.04 (0.03) | 0.27 | 0.05 (0.04) | | 0.26 | -0.66 (0.28) | **0.02** |
| iCV | -0.02 (0.04) | 0.62 | -0.00 (0.05) | | 0.96 | -0.23 (0.31) | 0.46 |
| cCV | -0.06 (0.04) | 0.08 | 0.04 (0.05) | | 0.41 | -0.80 (0.29) | **0.01** |
| Min DV | -0.06 (0.03) | 0.11 | 0.02 (0.05) | | 0.58 | -0.10 (0.30) | 0.73 |
| Max Latency | 0.03 (0.04) | 0.43 | -0.07 (0.04) | | 0.14 | 0.21 (0.31) | 0.51 |
| **Ischemic Stroke Cohort (N = 34, M = 45)** | | | | | | | |
|  | Beta (SE) | p | Beta (SE) | p | | Beta (SE) | p |
| Diff NPi | -0.02 (0.06) | 0.78 | 0.02 (0.06) | | 0.73 | 0.65 (0.44) | 0.14 |
| Diff Size | -0.02 (0.05) | 0.69 | 0.02 (0.06) | | 0.75 | 0.69 (0.40) | 0.09 |
| iSize | 0.06 (0.05) | 0.29 | 0.00 (0.06) | | 0.93 | -0.41 (0.40) | 0.32 |
| cSize | 0.03 (0.05) | 0.58 | -0.04 (0.05) | | 0.53 | -0.71 (0.40) | 0.08 |
| Min NPi | -0.06 (0.06) | 0.32 | 0.01 (0.06) | | 0.86 | 0.07 (0.45) | 0.88 |
| iNPi | -0.05 (0.05) | 0.32 | 0.00 (0.05) | | 0.95 | 0.28 (0.39) | 0.47 |
| cNPi | -0.06 (0.06) | 0.32 | 0.06 (0.06) | | 0.29 | 0.41 (0.44) | 0.75 |
| Avg NPi | -0.06 (0.06) | 0.26 | 0.01 (0.06) | | 0.81 | 0.25 (0.45) | 0.58 |
| Avg Size | 0.05 (0.05) | 0.34 | -0.01 (0.05) | | 0.79 | -0.59 (0.39) | 0.13 |
| Min CV | -0.00 (0.05) | 0.94 | 0.05 (0.06) | | 0.35 | -0.59 (0.41) | 0.16 |
| iCV | -0.00 (0.06) | 0.95 | 0.07 (0.06) | | 0.26 | -0.35 (0.43) | 0.42 |
| cCV | -0.01 (0.04) | 0.86 | 0.03 (0.05) | | 0.51 | -0.37 (0.34) | 0.29 |
| Min DV | -0.09 (0.06) | 0.13 | 0.05 (0.06) | | 0.43 | 0.07 (0.44) | 0.87 |
| Max Latency | 0.00 (0.05) | 0.94 | -0.07 (0.05) | | 0.20 | -0.24 (0.40) | 0.55 |
| **Intraparenchymal Hemorrhage Cohort (N = 19, M = 29)** | | | | | | | |
|  | Beta (SE) | p | Beta (SE) | p | | Beta (SE) | p |
| Diff NPi^*^ | -0.02 (0.04) | 0.61 | -0.03 (0.06) | | 0.68 | 0.19 (0.40) | 0.63 |
| Diff Size | 0.01 (0.06) | 0.82 | -0.01 (0.08) | | 0.88 | -0.07 (0.55) | 0.90 |
| iSize | -0.07 (0.04) | 0.16 | -0.10 (0.07) | | 0.18 | -0.11 (0.45) | 0.81 |
| cSize | -0.10 (0.05) | 0.05 | 0.00 (0.08) | | 0.99 | -1.06 (0.45) | **0.03** |
| Min NPi | -0.02 (0.05) | 0.64 | 0.06 (0.06) | | 0.31 | -0.62 (0.42) | 0.15 |
| iNPi | -0.04 (0.05) | 0.38 | 0.06 (0.06) | | 0.38 | -0.74 (0.45) | 0.11 |
| cNPi | -0.01 (0.05) | 0.82 | 0.05 (0.06) | | 0.47 | -0.49 (0.46) | 0.30 |
| Avg NPi | -0.03 (0.04) | 0.56 | 0.07 (0.06) | | 0.26 | -0.63 (0.41) | 0.14 |
| Avg Size | -0.12 (0.03) | **0.001** | -0.04 (0.07) | | 0.56 | -0.84 (0.30) | **0.02** |
| Min CV | -0.06 (0.05) | 0.21 | 0.06 (0.07) | | 0.45 | -0.73 (0.40) | 0.09 |
| iCV | -0.03 (0.05) | 0.59 | -0.05 (0.07) | | 0.50 | -0.10 (0.47) | 0.83 |
| cCV | -0.10 (0.06) | 0.10 | 0.06 (0.09) | | 0.52 | -1.15 (0.49) | **0.03** |
| Min DV | -0.04 (0.04) | 0.32 | 0.00 (0.06) | | 0.95 | -0.52 (0.38) | 0.19 |
| Max Latency | 0.06 (0.05) | 0.29 | -0.08 (0.08) | | 0.32 | 0.83 (0.45) | 0.08 |
| Abb.: Diff NPi-Absolute difference in left and right Neurologic Pupil Index; Diff Size-Absolute difference in left and right resting pupil size; IMW/CMW-Ipsilateral Midbrain Width/Contralateral Midbrain Width; M-Number of head Computed Tomography images; Min NPi-Minimum NPi of the left and right eye; N-Number of patients; NPi-Neurological Pupil index; SE-Standard Error. $\beta$ coefficients are reported as an increase in one unit of transformed pupil outcome using rank normalization. | | | | | | | |
