# Supplementary material for: Quantitative pupillometry and radiographic markers of intracranial midline shift: A pilot study
Source: Front Neurol. 2022 Dec 6;13:1046548. doi: 10.3389/fneur.2022.1046548 (PMC9763295; doi:10.3389/fneur.2022.1046548)
Supplement: Supplementary file 6 [file Table_6.docx]

|  | **MW** | | | **ML** | | **MW/ML** | |
| --- | --- | --- | --- | --- | --- | --- | --- |
| **Full Patient Cohort (N = 53, M = 74)** | | | | | | | |
|  | Beta (SE) | | p | Beta (SE) | p | Beta (SE) | p |
| Diff NPi | -0.02 (0.02) | | 0.47 | 0.03 (0.04) | 0.36 | -0.21 (0.22) | 0.34 |
| Diff Size | 0.01 (0.02) | | 0.73 | -0.02 (0.04) | 0.61 | 0.20 (0.23) | 0.39 |
| iSize | -0.01 (0.02) | | 0.78 | 0.01 (0.04) | 0.79 | -0.15 (0.23) | 0.51 |
| cSize | -0.01 (0.02) | | 0.70 | 0.01 (0.04) | 0.75 | -0.12 (0.23) | 0.62 |
| Min NPi | 0.01 (0.02) | | 0.76 | -0.03 (0.04) | 0.39 | 0.17 (0.22) | 0.46 |
| iNPi | -0.00 (0.02) | | 0.96 | -0.02 (0.04) | 0.52 | 0.07 (0.23) | 0.75 |
| cNPi | 0.02 (0.02) | | 0.47 | -0.03 (0.04) | 0.46 | 0.23 (0.22) | 0.23 |
| Avg NPi | 0.01 (0.02) | | 0.83 | -0.02 (0.04) | 0.54 | 0.14 (0.23) | 0.55 |
| Avg Size | -0.02 (0.02) | | 0.36 | 0.02 (0.04) | 0.59 | -0.26 (0.22) | 0.23 |
| Min CV | 0.02 (0.02) | | 0.35 | -0.01 (0.04) | 0.74 | 0.12 (0.23) | 0.61 |
| iCV | 0.02 (0.02) | | 0.40 | -0.05 (0.04) | 0.22 | 0.34 (0.23) | 0.15 |
| cCV | 0.01 (0.02) | | 0.61 | -0.00 (0.04) | 0.97 | 0.00 (0.23) | 0.99 |
| Min DV | -0.01 (0.02) | | 0.78 | -0.02 (0.04) | 0.52 | 0.05 (0.22) | 0.82 |
| Max Latency | -0.03 (0.02) | | 0.19 | 0.02 (0.04) | 0.66 | -0.28 (0.23) | 0.21 |
| **Ischemic Stroke Cohort (N = 34, M = 45)** | | | | | | | |
|  | Beta (SE) | p | | Beta (SE) | p | Beta (SE) | p |
| Diff NPi | 0.00 (0.04) | | 1.00 | 0.08 (0.05) | 0.13 | -0.36 (0.32) | 0.27 |
| Diff Size | -0.00 (0.03) | | 0.93 | 0.00 (0.05) | 0.97 | 0.15 (0.30) | 0.63 |
| iSize | 0.03 (0.04) | | 0.48 | 0.04 (0.05) | 0.38 | -0.21 (0.30) | 0.49 |
| cSize | -0.00 (0.03) | | 0.96 | 0.05 (0.05) | 0.33 | -0.20 (0.29) | 0.49 |
| Min NPi | -0.02 (0.04) | | 0.64 | -0.06 (0.05) | 0.21 | 0.35 (0.32) | 0.29 |
| iNPi | -0.02 (0.03) | | 0.56 | -0.04 (0.05) | 0.37 | 0.34 (0.28) | 0.24 |
| cNPi | 0.00 (0.04) | | 0.93 | -0.05 (0.05) | 0.32 | 0.43 (0.31) | 0.18 |
| Avg NPi | -0.02 (0.04) | | 0.62 | -0.05 (0.05) | 0.34 | 0.35 (0.32) | 0.28 |
| Avg Size | 0.01 (0.03) | | 0.67 | 0.05 (0.05) | 0.28 | -0.22 (0.29) | 0.45 |
| Min CV | 0.02 (0.04) | | 0.54 | -0.06 (0.05) | 0.26 | 0.28 (0.31) | 0.37 |
| iCV | 0.03 (0.04) | | 0.44 | -0.06 (0.05) | 0.21 | 0.44 (0.31) | 0.16 |
| cCV | 0.01 (0.03) | | 0.69 | -0.01 (0.04) | 0.76 | 0.11 (0.25) | 0.68 |
| Min DV | -0.02 (0.04) | | 0.64 | -0.05 (0.05) | 0.38 | 0.23 (0.33) | 0.49 |
| Max Latency | -0.03 (0.03) | | 0.42 | 0.04 (0.05) | 0.41 | -0.42 (0.29) | 0.15 |
| **Intraparenchymal Hemorrhage Cohort (N = 19, M = 29)** | | | | | | | |
|  | Beta (SE) | p | | Beta (SE) | p | Beta (SE) | p |
| Diff NPi^*^ | -0.04 (0.03) | | 0.18 | -0.02 (0.05) | 0.65 | -0.11 (0.29) | 0.70 |
| Diff Size | 0.01 (0.03) | | 0.88 | -0.05 (0.07) | 0.48 | 0.22 (0.37) | 0.56 |
| iSize | -0.05 (0.03) | | 0.10 | -0.02 (0.06) | 0.69 | -0.16 (0.35) | 0.64 |
| cSize | -0.02 (0.04) | | 0.49 | -0.03 (0.07) | 0.67 | -0.09 (0.39) | 0.83 |
| Min NPi | 0.03 (0.03) | | 0.24 | 0.02 (0.05) | 0.67 | 0.08 (0.29) | 0.79 |
| iNPi | 0.03 (0.03) | | 0.33 | 0.01 (0.05) | 0.90 | 0.12 (0.30) | 0.69 |
| cNPi | 0.03 (0.03) | | 0.36 | 0.02 (0.05) | 0.75 | 0.04 (0.31) | 0.90 |
| Avg NPi | 0.03 (0.03) | | 0.25 | 0.02 (0.05) | 0.72 | 0.08 (0.28) | 0.77 |
| Avg Size | -0.10 (0.02) | | **<0.001** | 0.00 (0.06) | 1.00 | -0.56 (0.29) | 0.07 |
| Min CV | 0.02 (0.03) | | 0.50 | 0.06 (0.06) | 0.34 | -0.20 (0.36) | 0.64 |
| iCV | 0.02 (0.03) | | 0.46 | -0.02 (0.06) | 0.75 | 0.30 (0.36) | 0.41 |
| cCV | 0.02 (0.04) | | 0.59 | 0.02 (0.08) | 0.81 | -0.04 (0.44) | 0.93 |
| Min DV | 0.01 (0.02) | | 0.60 | 0.02 (0.05) | 0.69 | -0.04 (0.28) | 0.89 |
| Max Latency | -0.04 (0.03) | | 0.24 | -0.01 (0.07) | 0.86 | -0.19 (0.37) | 0.61 |
| Abb.: Diff NPi-Absolute difference in left and right Neurologic Pupil Index; Diff Size-Absolute difference in left and right resting pupil size; Min NPi-Minimum NPi of the left and right eye; M-Number of head Computed Tomography images; MW-Midbrain Width; ML-Midbrain Length; MW/ML-Midbrain Width/Midbrain Length; N-Number of patients; NPi-Neurological Pupil index; SE-Standard Error. $\beta$ coefficients are reported as an increase in one unit of transformed pupil outcome using rank normalization. | | | | | | | |

**Supplementary Table 6**. Unadjusted Models Accounting for Inter-Patient Correlation (cont)
