# Supplementary material for: Quantitative pupillometry and radiographic markers of intracranial midline shift: A pilot study
Source: Front Neurol. 2022 Dec 6;13:1046548. doi: 10.3389/fneur.2022.1046548 (PMC9763295; doi:10.3389/fneur.2022.1046548)
Supplement: Supplementary file 7 [file Table_7.docx]

**Supplementary Table 7**. Univariate Models Accounting for Inter-Patient Correlation (cont)

| **Full Patient Cohort (N = 53, M = 74)** | | |
| --- | --- | --- |
|  | **Min IA** | |
|  | Beta (SE) | p |
| Diff NPi | -0.01 (0.01) | 0.92 |
| Diff Size | 0.00 (0.01) | 0.87 |
| iSize | 0.00 (0.01) | 0.17 |
| cSize | 0.00 (0.01) | 0.48 |
| Min NPi | 0.00 (0.01) | 0.78 |
| iNPi | 0.00 (0.01) | 0.91 |
| cNPi | 0.00 (0.01) | 0.60 |
| Avg NPi | 0.00 (0.01) | 0.70 |
| Avg Size | 0.00 (0.01) | 0.17 |
| Min CV | 0.00 (0.01) | 0.41 |
| iCV | 0.00 (0.01) | 0.61 |
| cCV | 0.00 (0.01) | 0.54 |
| Min DV | 0.00 (0.01) | 0.93 |
| Max Latency | -0.01 (0.01) | 0.31 |
| **Ischemic Stroke Cohort (N = 34, M = 45)** | | |
|  | Beta (SE) | p |
| Diff NPi | 0.01 (0.01) | 0.79 |
| Diff Size | 0.01 (0.01) | 0.77 |
| iSize | 0.01 (0.01) | 0.04 |
| cSize | 0.01 (0.01) | 0.12 |
| Min NPi | -0.01 (0.01) | 0.27 |
| iNPi | -0.01 (0.01) | 0.15 |
| cNPi | -0.00 (0.01) | 0.65 |
| Avg NPi | -0.01 (0.01) | 0.21 |
| Avg Size | 0.01 (0.01) | 0.04 |
| Min CV | 0.01 (0.01) | 0.57 |
| iCV | -0.00 (0.01) | 0.95 |
| cCV | 0.01 (0.00) | 0.36 |
| Min DV | -0.00 (0.01) | 0.52 |
| Max Latency | -0.00 (0.01) | 0.56 |
| **Intraparenchymal Hemorrhage Cohort (N = 19, M = 29)** | | |
|  | Beta (SE) | p |
| Diff NPi^*^ | -0.00 (0.01) | 0.76 |
| Diff Size | 0.00 (0.01) | 0.96 |
| iSize | 0.00 (0.01) | 0.63 |
| cSize | -0.00 (0.01) | 0.82 |
| Min NPi | 0.01 (0.01) | 0.37 |
| iNPi | 0.01 (0.01) | 0.42 |
| cNPi | 0.00 (0.01) | 0.74 |
| Avg NPi | 0.00 (0.01) | 0.28 |
| Avg Size | 0.00 (0.01) | 0.98 |
| Min CV | 0.01 (0.01) | 0.52 |
| iCV | 0.01 (0.01) | 0.35 |
| cCV | 0.00 (0.01) | 0.77 |
| Min DV | 0.00 (0.01) | 0.61 |
| Max Latency | -0.01 (0.01) | 0.39 |
| Abb.: Diff NPi-Absolute difference in left and right Neurologic Pupil Index; Diff Size-Absolute difference in left and right resting pupil size; IA-Interpeduncular Angle; M-Number of head Computed Tomography images; Min NPi-Minimum NPi of the left and right eye. N-Number of patients; NPi-Neurological Pupil index; SE-Standard Error. $\beta$ coefficients are reported as an increase in one unit of transformed pupil outcome using rank normalization. | | |
