# Supplementary material for: Quantitative pupillometry and radiographic markers of intracranial midline shift: A pilot study
Source: Front Neurol. 2022 Dec 6;13:1046548. doi: 10.3389/fneur.2022.1046548 (PMC9763295; doi:10.3389/fneur.2022.1046548)
Supplement: Supplementary file 8 [file Table_8.docx]

**Supplementary Table 8.** Exploratory multivariable model of Diff NPi and Radiographic Markers in patients with any hemorrhage present on imaging (primary or secondary) and patients with no hemorrhage on imaging.

|  | Beta (SE) | p |
| --- | --- | --- |
| **Full Patient Cohort (N = 34*, M = 45)** | | |
| MLS-SP | 0.01 (0.04) | 0.69 |
| PGS | 0.07 (0.06) | 0.24 |
| IMW/CMW | 0.65 (0.37) | 0.08 |
| Age | 0.01 (0.01) | 0.46 |
| Lesion volume | 0.00 (0.00) | 0.04 |
| GCS | -0.00 (0.05) | 0.98 |
| Osmotic Medications | 0.22 (0.31) | 0.48 |
| **Patients with any Hemorrhage on scan (Primary or Secondary) (N = 15, M = 20)** | | |
| MLS-SP | -0.03 (0.12) | 0.80 |
| PGS | 0.09 (0.19) | 0.64 |
| IMW/CMW | 0.86 (0.80) | 0.31 |
| Age | 0.00 (0.03) | 0.93 |
| Lesion volume | 0.00 (0.00) | 0.95 |
| GCS | 0.05 (0.11) | 0.67 |
| Osmotic Medications | 0.11 (0.75) | 0.89 |
| **Patients with No Hemorrhage on scan (N = 24, M = 25)** | | |
| MLS-SP | -0.02 (0.07) | 0.77 |
| PGS | 0.30 (0.12) | **0.02** |
| IMW/CMW | 0.70 (0.70) | 0.44 |
| Age | -0.01 (0.02) | 0.64 |
| Lesion volume | 0.00 (0.00) | 0.09 |
| GCS | 0.04 (0.09) | 0.67 |
| Osmotic Medications | 0.24 (0.68) | 0.73 |
| *Five patients initially did not have hemorhage transformation. Abb.: GCS -Glasgow Coma Scale; IMW/CMW-Ratio of Ipsilateral Midbrain Width and Contralateral Midbrain Width; MLS-SP-Midline Shift at Septum Pellucidum; PGS-Pineal Gland Shift | | |
