# Supplementary material for: Quantitative pupillometry and radiographic markers of intracranial midline shift: A pilot study
Source: Front Neurol. 2022 Dec 6;13:1046548. doi: 10.3389/fneur.2022.1046548 (PMC9763295; doi:10.3389/fneur.2022.1046548)
Supplement: Supplementary file 9 [file Table_9.docx]

**Supplementary Table 9.** Exploratory multivariable model of Diff Size and Radiographic Markers

|  | **Beta (SE)** | **p** |
| --- | --- | --- |
| **Full Patient Cohort (N = 53, M = 74)** | | |
| MLS-SP | 0.06 (0.04) | 0.12 |
| PGS | -0.05 (0.07) | 0.50 |
| IMW/CMW | 0.76 (0.42) | 0.07 |
| Age | 0.00 (0.01) | 0.43 |
| Lesion volume | 0.00 (0.00) | 0.21 |
| GCS | 0.03 (0.05) | 0.53 |
| Osmotic Medications | 0.12 (0.35) | 0.73 |
| **Ischemic Stroke Cohort (N = 34, M = 45)** | | |
| MLS-SP | -0.02 (0.06) | 0.68 |
| PGS | 0.06 (0.09) | 0.53 |
| IMW/CMW | 0.85 (0.51) | 0.11 |
| Age | 0.00 (0.01) | 0.74 |
| Lesion volume | 0.00 (0.00) | 0.89 |
| GCS | 0.03 (0.06) | 0.59 |
| Osmotic Medications | 0.64 (0.44) | 0.16 |
| **Intraparenchymal Hemorrhage Cohort (N = 19, M = 29)** | | |
| MLS-SP | 0.18 (0.05) | **<0.01** |
| PGS | -0.19 (0.11) | 0.10 |
| IMW/CMW | 0.39 (0.67) | 0.56 |
| Age | 0.00 (0.02) | 0.97 |
| Lesion volume | 0.01 (0.01) | 0.18 |
| GCS | 0.14 (0.09) | 0.14 |
| Osmotic Medications | -0.78 (0.51) | 0.14 |
| Abb.: GCS-Glasgow Coma Scale; IMW/CMW-Ratio of Ipsilateral Midbrain Width and Contralateral Midbrain Width; MLS-SP-Midline Shift at Septum Pellucidum; PGS-Pineal Gland Shift | | |
