# Supplementary figures and images for: Quantitative pupillometry and radiographic markers of intracranial midline shift: A pilot study
Source: Front Neurol. 2022 Dec 6;13:1046548. doi: 10.3389/fneur.2022.1046548 (PMC9763295; doi:10.3389/fneur.2022.1046548)

## Slide 1
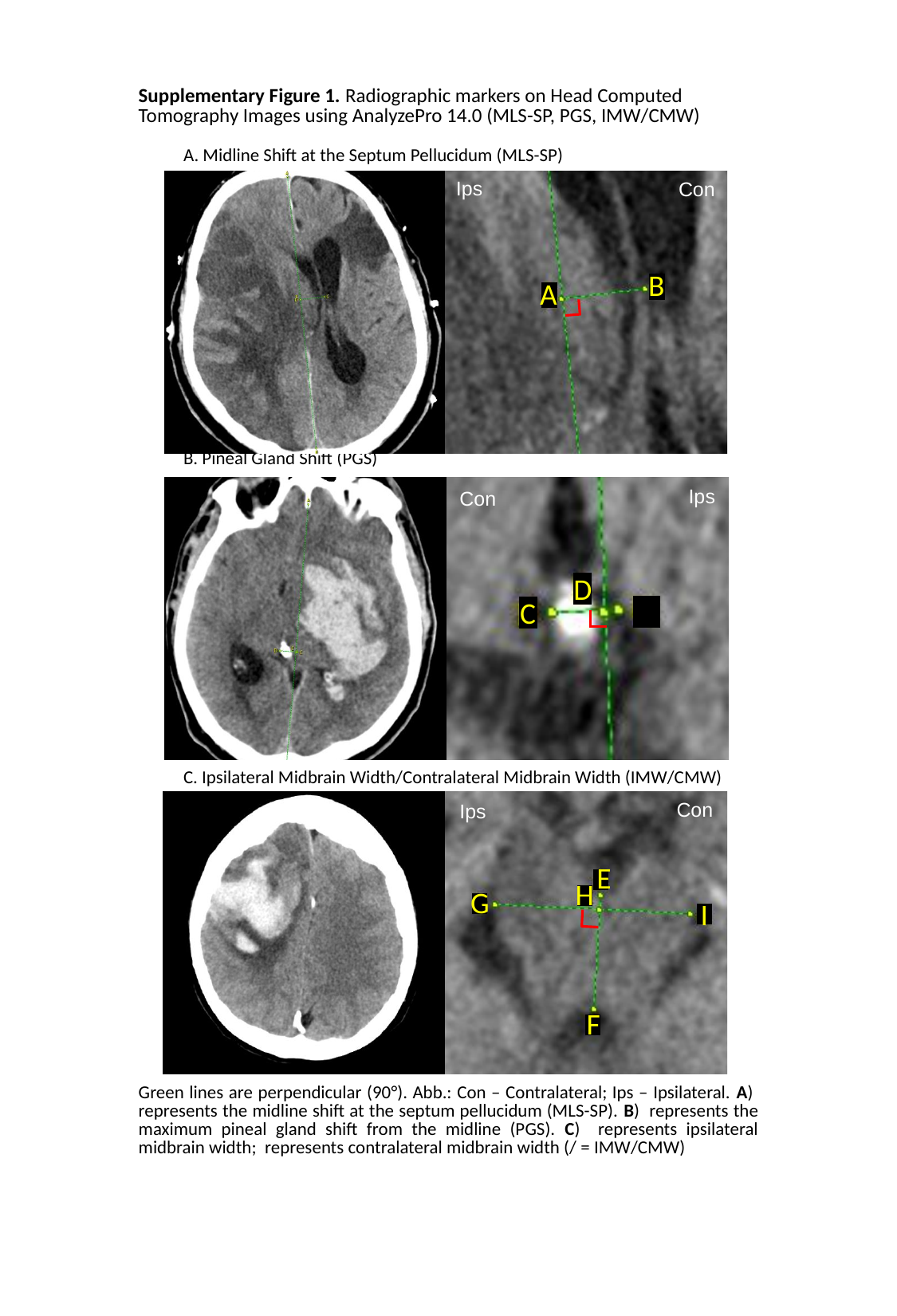

Ips
Con
Ips
Con
Con
Ips
B
A
D
C
E
H
G
I
F

Supplement: Supplementary file 10 [file Presentation_1.PPTX]

## Slide 1
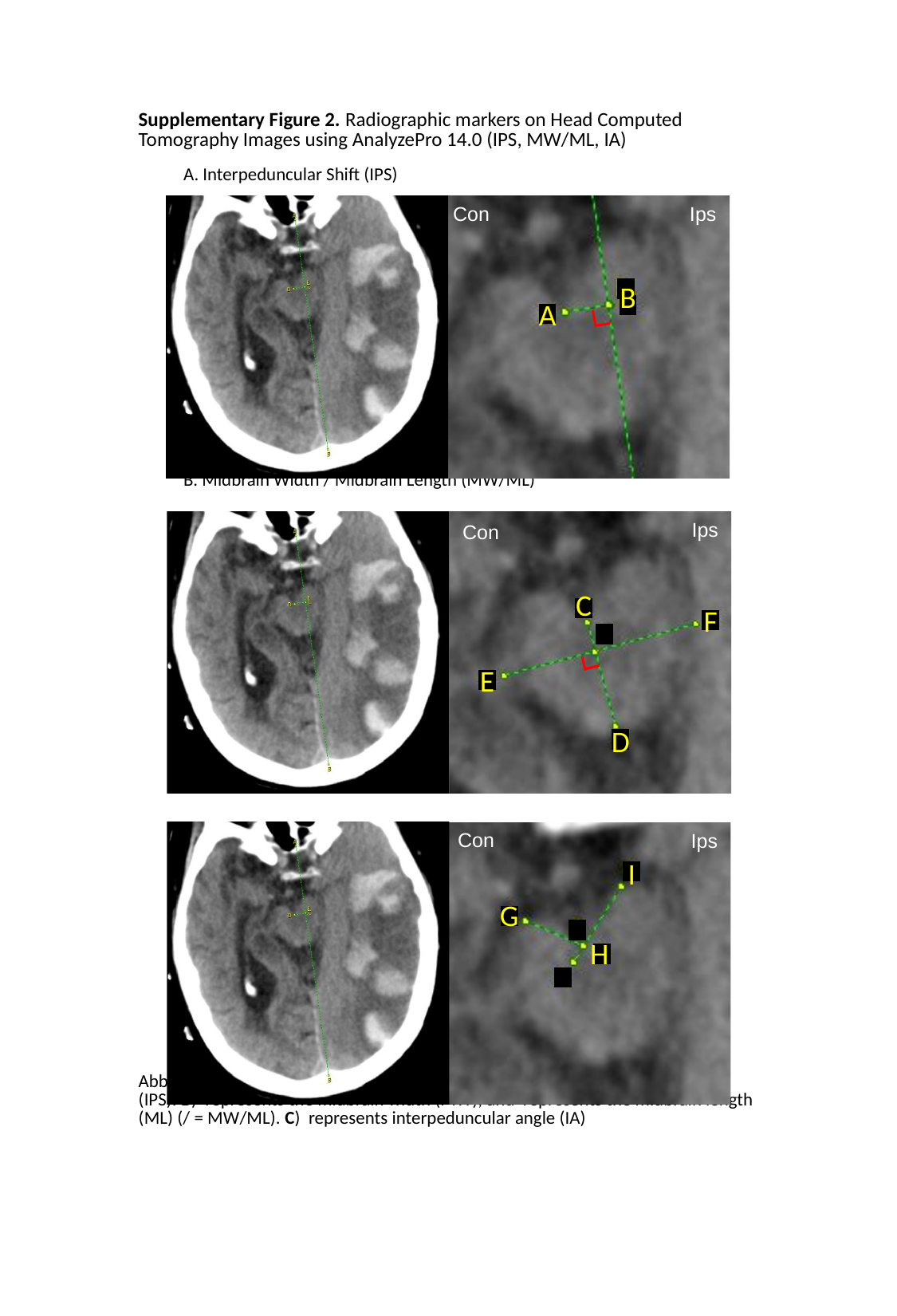

Con
Ips
Ips
Con
Con
Ips
B
A
C
F
E
D
I
G
H

Supplement: Supplementary file 11 [file Presentation_2.PPTX]
